# RNA CoMPASS: A Dual Approach for Pathogen and Host Transcriptome Analysis of RNA-Seq Datasets

**DOI:** 10.1371/journal.pone.0089445

**Published:** 2014-02-25

**Authors:** Guorong Xu, Michael J. Strong, Michelle R. Lacey, Carl Baribault, Erik K. Flemington, Christopher M. Taylor

**Affiliations:** 1 Department of Computer Science, University of New Orleans Lakefront, New Orleans, Louisiana, United States of America; 2 Department of Pathology, Tulane University, New Orleans, Louisiana, United States of America; 3 Department of Mathematics, Tulane University, New Orleans, Louisiana, United States of America; 4 Department of Microbiology, Immunology & Parasitology, Louisiana State University Health Sciences Center, New Orleans, Louisiana, United States of America; 5 Research Institute for Children, Children's Hospital of New Orleans, New Orleans, Louisiana, United States of America; Georgia Institute of Technology, United States of America

## Abstract

High-throughput RNA sequencing (RNA-seq) has become an instrumental assay for the analysis of multiple aspects of an organism's transcriptome. Further, the analysis of a biological specimen's associated microbiome can also be performed using RNA-seq data and this application is gaining interest in the scientific community. There are many existing bioinformatics tools designed for analysis and visualization of transcriptome data. Despite the availability of an array of next generation sequencing (NGS) analysis tools, the analysis of RNA-seq data sets poses a challenge for many biomedical researchers who are not familiar with command-line tools. Here we present RNA CoMPASS, a comprehensive RNA-seq analysis pipeline for the simultaneous analysis of transcriptomes and metatranscriptomes from diverse biological specimens. RNA CoMPASS leverages existing tools and parallel computing technology to facilitate the analysis of even very large datasets. RNA CoMPASS has a web-based graphical user interface with intrinsic queuing to control a distributed computational pipeline. RNA CoMPASS was evaluated by analyzing RNA-seq data sets from 45 B-cell samples. Twenty-two of these samples were derived from lymphoblastoid cell lines (LCLs) generated by the infection of naïve B-cells with the Epstein Barr virus (EBV), while another 23 samples were derived from Burkitt's lymphomas (BL), some of which arose in part through infection with EBV. Appropriately, RNA CoMPASS identified EBV in all LCLs and in a fraction of the BLs. Cluster analysis of the human transcriptome component of the RNA CoMPASS output clearly separated the BLs (which have a germinal center-like phenotype) from the LCLs (which have a blast-like phenotype) with evidence of activated MYC signaling and lower interferon and NF-kB signaling in the BLs. Together, this analysis illustrates the utility of RNA CoMPASS in the simultaneous analysis of transcriptome and metatranscriptome data. RNA CoMPASS is freely available at http://rnacompass.sourceforge.net/.

## Introduction

Through its capacity to delve deeply into the genetic composition of a biological specimen, next generation sequencing (NGS) technology presents an unprecedented approach to pathogen discovery in the context of human disease. This unbiased approach to identify undiscovered human disease causing pathogens has already shown promise, resulting in the discovery of a novel Merkel cell polyomavirus in Merkel cell carcinoma [1], for example. More recently, the discovery of an association between *Fusobacterium* and colorectal carcinoma was made using two different NGS approaches [2], [3]. These discoveries were facilitated by the use of computational subtraction approaches where reads aligning to reference genomes were subtracted from the sequence file leaving behind sequences from undiscovered organisms. Using this general approach, several groups, including ours, have previously reported computational pipelines for the analysis of exogenous sequences and for pathogen discovery [2], [4]–[8].

While current sequence-based computational subtraction pipelines are used solely for pathogen discovery, RNA CoMPASS, takes advantage of the richness of RNA-seq data to provide host transcript expression data in addition to pathogen analysis. This concept, recently coined “dual RNA-seq” by Westermann and colleagues [9] allows the user to simultaneously investigate cellular signaling pathways. It also allows the user to investigate associations between differences in cellular signaling pathways and the presence or absence of discovered pathogens. RNA CoMPASS leverages some of the most useful freely available tools and automates distribution of the computational burden over the available computing resources. It is designed to be deployable on either a local cluster or a grid environment managed by Portable Batch System (PBS) submission. RNA CoMPASS provides a web-based graphical user interface, making the program accessible to most biological researchers. Here we present RNA CoMPASS and demonstrate its utility in dual analysis of RNA-seq data sets from different B-cell types with different EBV infection status.

## Materials and Methods

### Sequence data acquisition

RNA-seq data sets from 22 Human B-Cell samples (lymphoblastoid cell lines [LCLs]) immortalized with Epstein-Barr Virus (EBV) were downloaded from the NCBI Sequence Read Archive (SRA010302). Samples were sequenced using an Illumina Genome Analyzer II machine running single end 50 base sequencing reactions. Similarly, 22 Human Burkitt's Lymphoma (BL) samples were obtained from the NCBI Sequence Read Archive (SRA048058). Samples were sequenced using an Illumina Genome Analyzer II machine running paired end 107 and 102 base sequencing reactions. The Akata RNA-seq data set was generated previously in our lab (SRA047981) [10]. The Akata sample was sequenced using an Illumina HiSeq instrument running paired end 100 base sequencing reactions. A list of all the samples used is provided in Table S1.

### RNA CoMPASS

RNA CoMPASS (RNA comprehensive multi-processor analysis system for sequencing) is a graphical user interface (GUI) based parallel computation pipeline for the analysis of both exogenous and human sequences from RNA-seq data. Several open source programs and a single commercial program are utilized in this automated pipeline. For the deduplication steps, an in-house de-duplication algorithm is used. Alignments to the reference genome are carried out using Novoalign V2.07.18 (www.novocraft.com) [-o SAM, default options] with a reference genome (e.g. human (hg19; UCSC)), splice junctions (which is generated using the make transcriptome application from Useq [11]; splice junction radius is set to the read length minus 4), and abundant sequences (which include sequence adapters, mitochondrial, ribosomal, enterobacteria phage phiX174, poly-A, and poly-C sequences). Human mapped reads are analyzed using SAMMate [12] to quantify gene expression and to generate genome coverage information. Nonmapped reads are separated following this alignment and subjected to consecutive BLAST V2.2.27 searches against the Human RefSeq RNA database (a final filtering step) and then to the NCBI NT database to identify reads corresponding to known exogenous organisms [13]. Results from the NT BLAST searches are filtered to eliminate matches with an E-value of less than 10e^−6^. The results are fed into MEGAN 4 V4.62.3 [14] for convenient visualization and taxonomic classification of BLAST search results.

### Statistics and Cluster analysis

Human transcript counts were imported into the R software environment and analyzed using the edgeR package [15]. Genes with low transcript counts (less than 1 CPM (count per million)) in the majority of samples were filtered out. The Manhattan (L-1) distance matrix for the samples was computed using the remaining transcript counts, and this was taken as input for hierarchical clustering using the Ward algorithm. After assigning each sample to one of two groups identified by hierarchical clustering (Human B-Cell or Burkitt's Lymphoma), the glmFit function was used to fit the mean log(CPM) for each group and likelihood ratio tests were used to identify those genes that were differentially expressed, with adjusted *P*<0.05 following the Benjamini-Hochberg correction for multiple testing. The fitted log(CPM) values for the subset of genes that were differentially expressed in the LCL samples relative to the BL samples were then clustered using the Euclidean distance and complete linkage algorithm to detect groups of co-expressed genes.

## Results

### RNA CoMPASS Architecture

**Figure 1:**
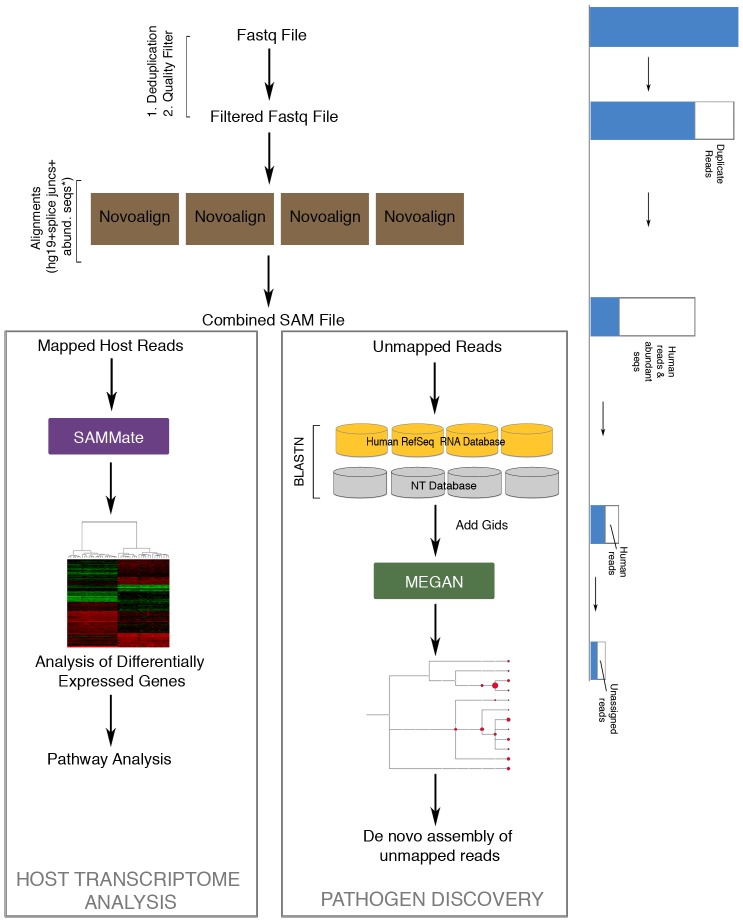
Schematic of RNA CoMPASS (RNA comprehensive multi-processor analysis system for sequencing) architecture. RNA CoMPASS is a graphical user interface (GUI) based parallel computation pipeline for the analysis of both exogenous and human sequences from RNA-seq data. It employs a commercial and several open-source programs to analyze RNA-seq data sets including Novoalign, SAMMate, BLAST, and MEGAN. Each step results in the subtraction of reads in order to further analyze the unmapped reads for pathogen discovery. The mapped reads are analyzed separately. The end result from this pipeline is pathogen discovery and host transcriptome analysis.

RNA CoMPASS facilitates the analysis of small and large RNA sequencing studies through an automated dataflow management and acceleration of processing via distributed computing over a cluster (Figure 1). It has the capability to analyze fastq sequence files generated from single-end, paired-end, and/or directional sequencing strategies. After an initial deduplication step, the first phase of RNA CoMPASS is to perform the alignment of millions of short reads against the host genome using an accurate aligner, Novoalign (http://www.novocraft.com/) [-o SAM, default options]. Any host genome can be uploaded to RNA CoMPASS. In our case, we used the human reference genome, hg19 (UCSC), plus splice junctions (which is generated using the make transcriptome application from Useq [11]; splice junction radius is set to the read length minus 4), and abundant sequences (which include sequence adapters, mitochondrial, ribosomal, enterobacteria phage phiX174, poly-A, and poly-C sequences). After alignment, Novoalign categorizes reads into four classes: uniquely mapped reads, repeat mapped reads, unmapped reads and quality controlled reads. Further processing is bifurcated into the analysis of endogenous sequences (uniquely mapped reads and repeat mapped reads) and the investigation of exogenous reads (unmapped reads) (Figure 1).

Endogenous sequence analysis is performed via the SAMMate transcript analysis software (Figure 1) [12]. A gene annotation file of interest is uploaded to facilitate the calculation of expression abundance scores for annotated genes and transcripts using the uniquely mapped reads (this includes spliced reads) and the best hits of repeat mapped reads from Novoalign. Gene expression is calculated using Reads/Fragments Per Kilobase of exon model per million Mapped reads (RPKM/FPKM) [16]. Isoform quantification is also computed via the RAEM algorithm [17] or the iQuant procedure [18] to estimate the relative isoform proportions and abundance scores. RNA CoMPASS also generates useful files for visualization of RNA-seq data. Read coverage files are produced in Wiggle format for coverage viewing in a genome browser and signal map files are produced with single base pair resolution which can be used with peak detection algorithms [12].

Exogenous sequence analysis proceeds concurrently with the endogenous analysis (Figure 1). Utilizing BLAST [19], unmapped reads are searched against the NCBI NT database for identification using an E-value of better than 10e^−6^. This process is extremely computationally intensive and is distributed across the computing cluster to minimize processing time and memory requirements. BLAST run time and memory requirements depend not only on the size of the database being searched but also on the number of input reads. The filtering of reads originating from the human genome prior to searching against the NCBI NT database is the first major step in managing this burden. Despite this step, we have discovered that many host reads remain unmapped and are subsequently identified by BLAST (since BLAST is substantially more permissive). To further reduce the computational burden incurred by BLASTing these unmapped host reads, RNA CoMPASS offers an optional stage prior to NT database BLASTing where the user can BLAST against a host transcript database. Because host transcript databases are much smaller than the NT database, host reads not aligned by Novoalign can be filtered out at lower computational cost than would otherwise be incurred by BLASTing these reads against the NT database.

After BLASTing against the NT database, taxonomic analysis is performed by importing the BLAST results into MEGAN [14]. To allow MEGAN to determine the taxon associated with each match, the NCBI taxon id number is appended to each BLAST hit. This is accomplished by looking up the GI accession number in the GI to TaxID file using a custom script. MEGAN then determines the taxon associated with matches based on the hit table using a lowest common ancestor algorithm. MEGAN categorizes the exogenous sequences and outputs an NCBI taxonomy tree. Each node of the output tree is labeled by a taxon and the size of a given node represents the number of reads assigned to that taxon. This provides the researcher with an overview of reads of possible exogenous origin. The researcher can then evaluate the exogenous sequence content in the context of their own biological knowledge of the experiment at hand. The researcher can also formulate hypotheses to test given the taxonomic classification displayed by MEGAN and then export all reads that were assigned to a specific taxon for further analysis. For example, the reads can be assembled into longer transcripts [20] using a de novo parallel sequence assembler. This provides the researcher with a broader view of the particular transcripts that were found within a given taxon. De novo assembly can be repeated for each taxon of interest and the researcher can search the longer assembled transcripts against the databases again to get more precise hits.

In RNA CoMPASS, we have implemented both the Java Parallel Processing Framework (JPPF) API and Portable Batch System (PBS) API in order to deploy it on either a small local cluster or a grid system managed by PBS submission. Our testing of RNA CoMPASS in both environments (data not shown for grid system) showed that our pipeline could efficiently analyze RNA-seq data sets achieving a significant speedup over analysis on a single machine. This will allow other investigators to use RNA CoMPASS on whichever type of computational environment they have access to. In our case, we employed RNA CoMPASS on a local 4-node cluster environment (Intel Xeon Mac Pros with 64–96 GB RAM).

### RNA CoMPASS performance

**Figure 2:**
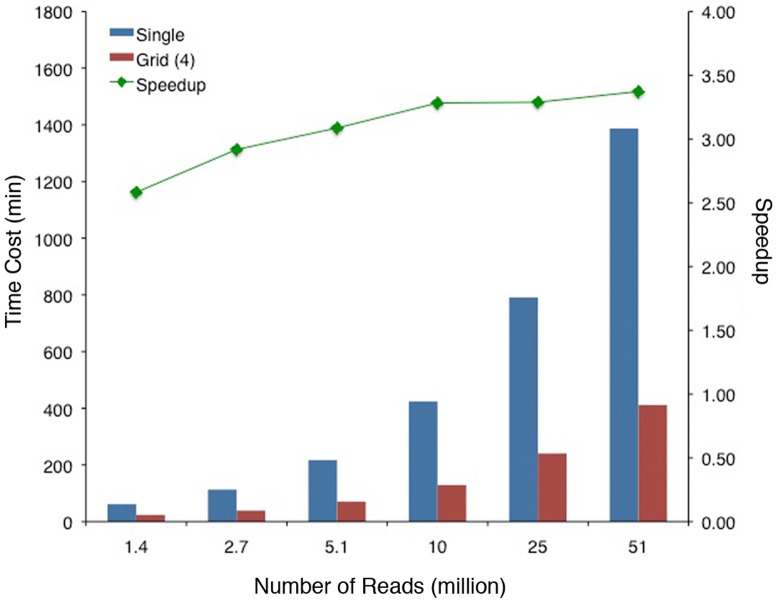
Performance Analysis of RNA CoMPASS. RNA CoMPASS was deployed on a local cluster and benchmarking was performed. An Akata RNA-seq data set was split into six files of varying sizes: 1–393.4 MB, 1,397,139 reads, 2–757 MB, 2,685,149 reads, 3–1.44 GB, 5,120,805 reads, 4–2.72 GB, 9,651,466 reads, 5–5.01 GB, 25,465,406 reads, sample 6–8.99 GB, 50,930,812 reads. Overall time was calculated for each file on a single machine (blue column) and on the local 4-node cluster (red column). Speedup time is represented as a green line.

To evaluate the performance of RNA CoMPASS on a cluster environment versus a single node environment, we benchmarked 6 RNA-seq data sets with incrementally varied file sizes on both a single machine and on a local cluster with 4 nodes. The 6 files used for this analysis were extracted from a previously generated RNA-seq data set from a BL cell line (1 sequence pair from the Akata RNA-seq data set) [10] and the file sizes varied from 1.4 to 51 million reads. All 6 samples, run on the single node or the 4-node cluster, were processed using identical parameters. As expected, run time increased with file size with the 51 million read file taking approximately 1,400 minutes on a single machine but only 400 minutes on the cluster (Figure 2). Speedup increased with file size (up to 3.4, Figure 2) supporting a benefit of a cluster environment for large-scale projects. Overall speedup was attributed primarily to the parallelization of the two more computationally intensive steps, Novoalign and BLAST (Figure S1).

### Pathogen Discovery and Analysis

To test the utility of RNA CoMPASS to identify pathogens within biological specimens we used RNA-seq data sets from two distinct B-cell types, lymphoblastoid cell lines (LCLs) and Burkitt's lymphoma (BL) samples. LCLs are not tumor cells but have instead been immortalized by infection with EBV. In contrast, the Burkitt's lymphoma cells lines are tumor cell lines, some of which underwent tumorigenesis in part through natural infection with EBV. Notably, however, although some Burkitt's lymphoma cell lines are infected with EBV, the EBV gene expression pattern and the cell phenotype of Burkitt's lymphomas and LCLs are distinct.

**Figure 3:**
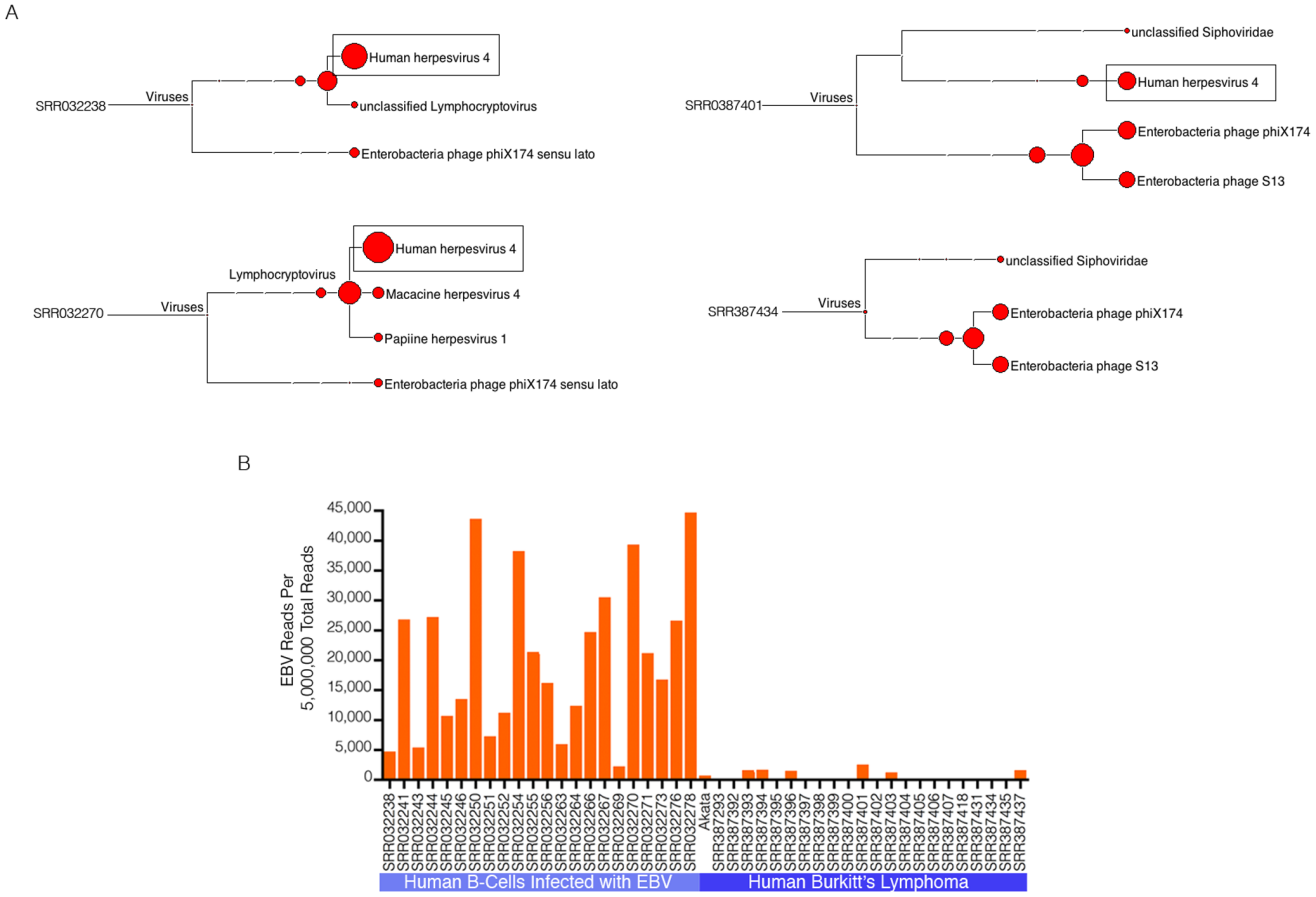
Detection of EBV in Human B-Cells using RNA CoMPASS. Analysis of all 45 single-end RNA-seq data sets (22-Lymphoblastoid cell lines, 23-Burkitt's lymphomas) were analyzed using RNA CoMPASS. (A) The virome branch of the taxonomy trees for two representative LCLs and Burkitt's lymphomas were generated using the metagenome analysis tool, MEGAN 4. (B) EBV reads were quantified in all 45 RNA-seq data sets and are represented as per 5,000,000 total sequence reads.

Single-end RNA-seq data sets from 45 B-cell lines (22-Lymphoblastoid cell lines, 23-Burkitt's lymphomas) were analyzed using RNA CoMPASS (Table S1). Most samples contained relatively low numbers of non-human viral reads (e.g. enterobacteria phage) that most likely represent environmental contamination (Figure 3A). EBV was the primary mammalian virus detected in these samples (displayed as Human herpesvirus 4 in examples shown in Figure 3A). Nevertheless, related viruses were sometimes displayed in the MEGAN output such as that for sample SRR032270 where 88 reads were classified as Macacine herpesvirus 4 reads and 32 were classified as Papiline herpesvirus 1 (Figure 3A). Further analysis of these reads using manual BLAST revealed that EBV ranked among the top 2 hits suggesting that these reads are likely EBV but were misclassified. Most importantly, RNA CoMPASS identified all 22 LCLs and 7 of the 23 Burkitt's lymphoma samples as being positive for EBV (Figure 3B).

**Figure 4:**
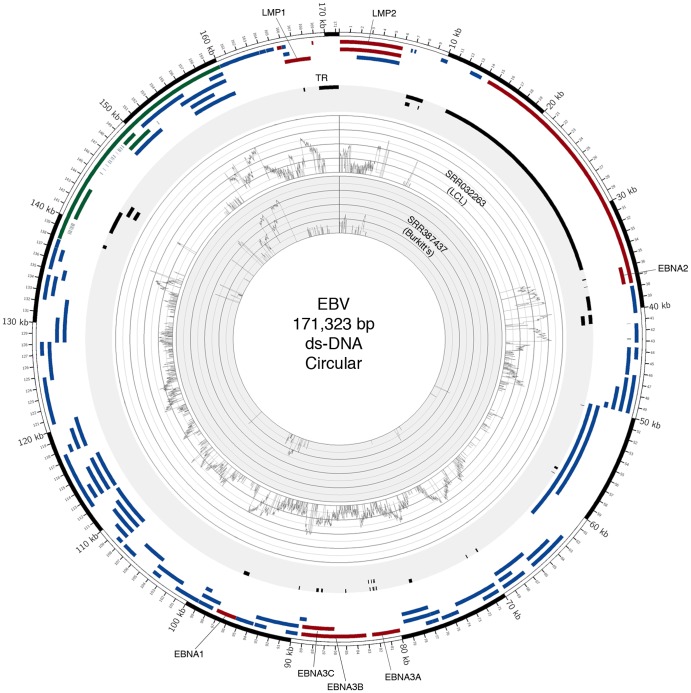
Circos plot of two EBV samples shows distinct gene expression. An annotated Circos plot depicts the EBV read coverage across the EBV genome of two samples. The graph displays the number of reads mapped to each nucleotide position of the genome and are depicted in log scale. Blue features represent lytic genes, red features represent latency genes, green features represent potential non-coding genes, and black features represent non-gene features (e.g. repeat regions and origins of replication).

As expected, EBV gene expression in LCLs is generally more robust and shows the expression profile expected in this cell type with all the latent proteins including EBNA 1,2, and 3 and LMP 1 and 2 being expressed (Figure 4). In contrast, the BL samples showed the expected more restricted gene expression pattern (referred to as type 1 latency) with regions in the BamHI A and the EBNA 1 loci showing coverage (Figure 4).

### Host Transcriptome Analysis

**Figure 5:**
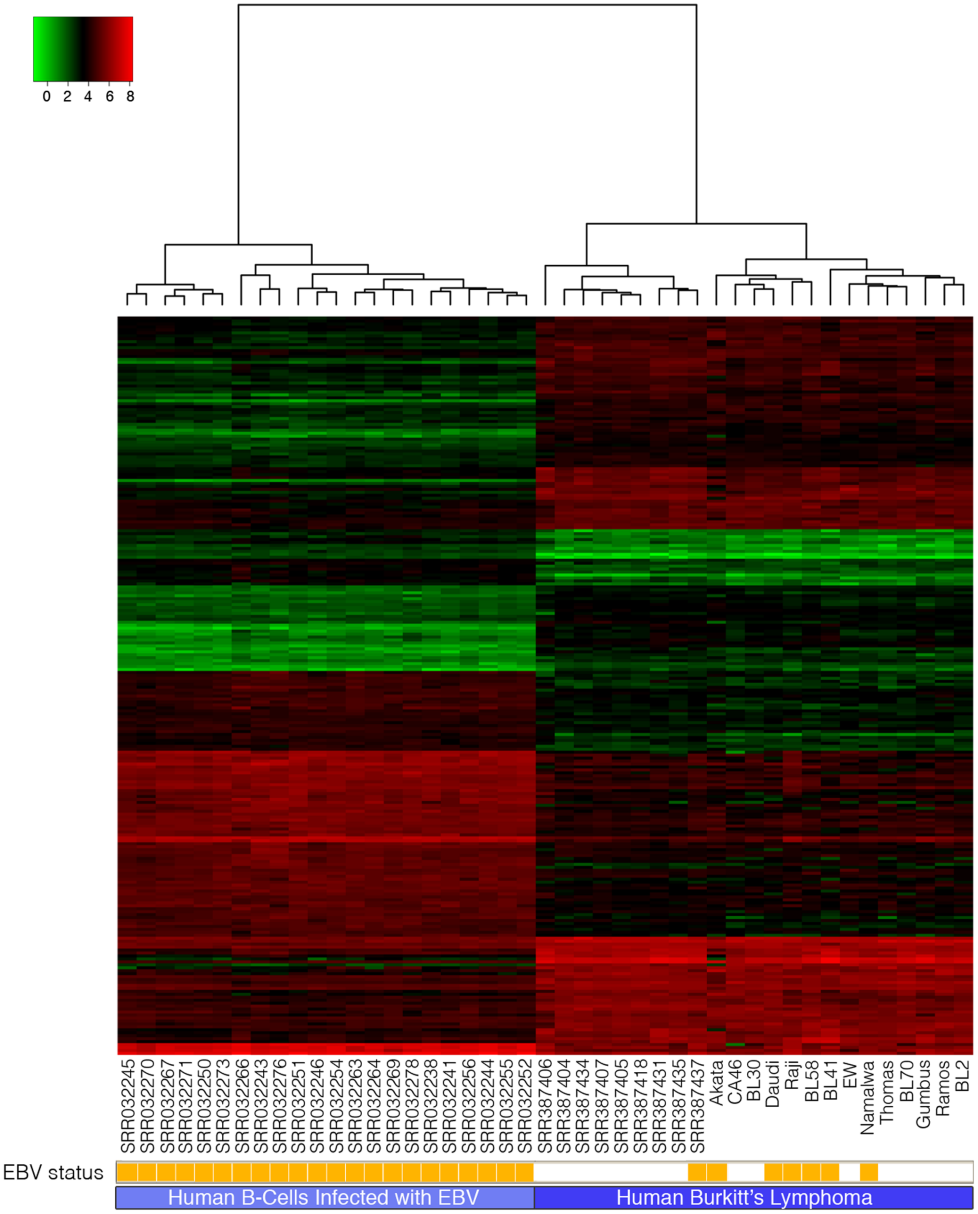
Heat Map representing Human B-Cells analyzed using RNA CoMPASS. Human transcript counts from the 45 B-cell samples were imported into the R software environment and analyzed using the edgeR package [15]. Genes with low transcript counts (less than 1 CPM (count per million)) in the majority of samples were filtered out. The Manhattan (L-1) distance matrix for the samples was computed using the remaining transcript counts, and this was taken as input for hierarchical clustering using the Ward algorithm. After assigning each sample to one of two groups identified by hierarchical clustering (Human B-Cell or Burkitt's Lymphoma), the glmFit function was used to fit the mean log(CPM) for each group and likelihood ratio tests were used to identify those genes that were differentially expressed, with adjusted *P*<0.05 following the Benjamini-Hochberg correction for multiple testing. The fitted log(CPM) values for the subset of genes that were differentially expressed in the LCL samples relative to the Burkitt's lymphoma samples were then clustered using the Euclidean distance and complete linkage algorithm to detect groups of co-expressed genes. The expression heat map displays the top 250 differentially expressed genes.

The host transcriptome analysis component of RNA CoMPASS generates gene expression output files that can be used for cluster and pathway analysis. Gene expression output from RNA CoMPASS analysis of the 22 LCL and 23 BL samples was subjected to hierarchical clustering and differential gene expression analysis (Table S2 contains the list of the top 250 differentially expressed genes and Table S3 contains the list of all the differentially expressed genes). Using the Ward criterion, the samples separated in two well defined clusters with one cluster representing the LCL phenotype and the other representing the BL phenotype (Figure 5). Furthermore, within the BL cluster, biopsies separated from the cell lines, possibly caused by the contribution of stromal signals in the biopsies ads/or by genetic drift in the cell lines.

To investigate differences in LCLs compared to BLs, Ingenuity Pathway Analysis software (IPA: Ingenuity Systems) was used to assist in the analysis of signaling pathways and molecular functions associated with the differentially expressed cellular genes. Upstream regulator analysis within IPA predicted activation of MYC (z-score: 3.375), MYCN (z-score: 2.813), MAPK9 (z-score: 2.414), and MAPK1 (z-score: 2.138) pathways with an inhibition of Interferon alpha (z-score: −2.916), interferon gamma (z-score: −2.788), NF-kB (z-score: −2.746), interferon alpha-2 (z-score: −2.723), and interferon lambda (z-score: −2.000) pathways in BL relative to LCL samples (Figure 5, Figures S2 and S3). TCF3 (5.4-fold) and TOP2A (9.0-fold) were both increased in BLs relative to LCLs.

## Discussion

RNA CoMPASS is designed to take advantage of several open source programs in order to streamline and accelerate RNA-seq data analysis. RNA CoMPASS helps the researcher to manage the computational burden of processing large sets of RNA-Seq data by parallelizing the most compute intensive steps of the process and automatically managing files through each step of the pipeline. The simultaneous analysis of the host transcriptome along with the discovery of pathogens allows investigators to not only detect pathogens but also study the relationship between the pathogen and host transcription.

In RNA CoMPASS, we have implemented both the Java Parallel Processing Framework (JPPF) API and Portable Batch System (PBS) API in order to deploy it on either a small local cluster or a grid system managed by PBS submission. Our testing of RNA CoMPASS in both environments showed that our pipeline could efficiently analyze RNA-seq data sets achieving a significant speedup over analysis on a single machine. This will allow other investigators to use RNA CoMPASS on whichever type of computational environment they have access to. In our case, we employed RNA CoMPASS on a local 4-node cluster environment (Intel Xeon Mac Pros with 64-96GB RAM) which achieves a speedup approaching the theoretical limit of 4 by splitting the computational tasks over 4 machines as the file size increases. This speedup of a 4 node cluster serves as a proof of principle that an even greater speedup could be obtained using a significant computational cluster involving hundreds of nodes. A recently published study [21] also outlines a different approach using the Bowtie aligner (which is significantly faster than novoalign) to align against the human plus virus genomes. As an alternative approach, they use BLASTing of de novo assembled reads instead of all unaligned reads. These are computationally more efficient approaches but the first method is constrained by index size limitations, which preclude the inclusion of a broad array of organisms such as bacteria and fungi, for example. In contrast, the BLAST approach of RNA CoMPASS surveys the entire NT database. The blasting of only de novo assembled reads would also significantly speed up our approach, however, BLASTing raw reads allows us to quantify relative levels of each exogenous agent found, which is an important read-out for these studies.

Though the BLASTing step of RNA CoMPASS incurs moderate limits on the size of input file that can be processed (depending on access to a large computational cluster), it allows for a more comprehensive analysis. In previous work, we would that sampling 10 million reads from an RNA sequencing experiment is likely to be well beyond the number needed to detect meaningful levels of exogenous agents [22]. A future enhancement of RNA CoMPASS will be to leverage this result and first align all reads from a sample for analysis of human reads, but then to carry forward only an adequately sized sample of unmapped reads through the more computationally burdensome analysis of exogenous agents (BLASTing). Future implementations of RNA CoMPASS are also under development which will leverage large computing clouds (like Amazon EC2) and will also provide the option of using alternative aligners such as Bowtie or STAR to significantly speed up the alignment process.

We used LCLs and BL samples to evaluate the pathogen and host transcriptome analysis arms of RNA CoMPASS because of their differences in phenotypes. The LCLs were generated by infecting human B-cells with EBV, which typically display an activated B-cell like phenotype (type III latency – expressing all 9 EBV latency genes (LMP1, LMP2A, LMP2B, EBNA1, EBNA-LP, EBNA2, EBNA3A, EBNA3B, and EBNA3C) and BART transcripts). The BL samples typically display a germinal center-like phenotype (type I latency – expressing EBNA1 and BART transcripts). The entire sequence file for all samples was used as input for RNA CoMPASS. Although the BL samples were sequenced using a paired-end approach, only one of the reads from each pair was analyzed in order to remain consistent among all samples and because a single-end read should provide sufficient evidence for pathogen discovery.

RNA CoMPASS discovered a significantly larger proportion of EBV (Human Herpesvirus 4) reads within the LCLs as compared to the BLs. This is an anticipated result, which validates the usage of RNA CoMPASS to interrogate genetic material of exogenous origin.

One of the most highly active pathways within BLs is the MYC pathway. In our study, the MYC and MYCN pathways were predicted to be the top two activated pathways in BLs relative to LCLs according to IPA's upstream regulator analysis. Several MYC targets have been reported in the literature [23]–[27] and we see many of these targets regulated in our study including the MYC-induced genes, BUB1, CENPF, CCNB1, PLK1, PCNA, AURKB; and the MYC-repressed genes: STAT1, IL10RA, and HLA-DRA. In addition, the MYC pathway has emerged as one of the central regulators of cell growth and ribosome biogenesis by inducing several genes encoding ribosomal proteins [28]. In our study, we observe that the top differentially expressed gene targets of MYC and MYCN are related to ribosomal protein synthesis (NCL, RPL30, RPL37, RPS20, and RPL3).

Transcriptome analysis has shown that the MYC signature is the hallmark signaling difference between Burkitt's lymphomas and diffuse large B-cell lymphomas with: upregulation of MYC-target genes and downregulation of genes involved in the NF-kB and interferon responses [23], [24], [27]. This hallmark signaling is recapitulated in our study using transcriptome analysis of BL and LCL samples. Taken together, the results presented here as well as from others indicate that a single master transcriptional pathway, MYC, mainly governs the growth potential of BLs with the help of other oncogenes as cofactors [27].

On the other hand, the most highly inhibited pathways within BLs compared to LCLs were the interferon response pathway and the NF-kB pathway. The NF-kB pathway has been shown to play a vital role in EBV's ability to transform naïve B-cells as the EBV transforming latency protein, LMP1 continuously activates NF-kB [29], [30]. Among other genes, we observe an increased expression of antigen presentation molecules in LCLs relative to BLs, possibly through the LMP1/NF-kB pathway [29]. The inhibition of the interferon response pathways seen in BLs lends to the overexpression of MYC contributing to immune escape through repression of the interferon response [31].

A few other noteworthy genes that were observed as being differentially expressed include TCF3 and TOP2A, both of which have increased expression in BLs relative to LCLs. In a recent study using RNA-seq with RNA interference screening of BLs, Schmitz et al was able to identify mutations affecting the transcription factor TCF3 [32]. TCF3 has been shown to activate the pro-survival phosphatidylinositol-3-OH (PI(3)) kinase in part by augmenting B-cell receptor signaling [32]. The authors suggest that the MYC and PI(3) kinase pathways may act synergistically in BL oncogenesis, and that the PI(3) kinase pathway may be a new target for drug development [32].

The other molecule, TOP2A has been shown to determine anthracycline-based drug (e.g. doxorubicin) response *in vitro* and *in vivo* [33]. Dose intensity of doxorubicin was evaluated by Kwak and colleagues in a retrospective analysis of 115 patients with diffuse large B-cell lymphoma [34]. The outcome of this study determined that doxorubicin should be used for the treatment of aggressive non-Hodgkin's lymphomas and dose intensity of doxorubicin was a key factor in predicting patient survival. Further, a meta-analysis of published randomized controlled trials comparing chemotherapy regimens incorporating doxorubicin at a high dose with standard CHOP therapy was conducted and their conclusions were consistent with the Kwak and colleagues study [35]. Altogether, levels of TOP2A in BLs are elevated relative to immortalized B cells and that high dose doxorubicin in addition to standard CHOP therapy may improve Burkitt's lymphoma patient outcome through TOP2A mediated doxorubicin response.

## Conclusion

In summary, our results demonstrate the utility of RNA CoMPASS in analyzing large sequence datasets for the discovery of pathogens and host transcriptome analysis. The use of this pipeline is expected to enable more researchers to enter the filed of RNA Sequencing and to yield novel associations between pathogens and human diseases with important medical implications. This study shows the disparate expression profiles between Lymphoblastoid cell lines and the Burkitt's lymphoma samples thereby exhibiting the ability of RNA CoMPASS to analyze endogenous sequencing. RNA CoMPASS is publically available under the GPL: http://rnacompass.sourceforge.net. Installation guidelines, supporting documentation, along with example data are available from the sourceforge repository. The most up to date source code is available from the sourceforge site; the version of the source code at the time of publication is included in Software S1: Source Code.

We are planning to implement the Circos plot capability for discovered pathogens as well as clustering analysis for host gene expression in later versions of RNA CoMPASS. These improvements will further streamline and complement the analysis of RNA-seq data in the discovery and analysis of pathogens associated with malignancies. In addition, we are investigating ways to further improve the speedup of the pipeline.

## Supporting Information

Figure S1 **Performance of RNA CoMPASS based on individual tasks.** The six Akata RNA-seq data set files used previously were benchmarked on completion of individual tasks and represented in the graphs. Runs on a single node are represented using blue columns while runs on a 4-node cluster are represented using red columns. The green line represents speedup time between the single node and 4-node environment. Note in particular that speedup of the BLAST portion of RNA CoMPASS and overall speedup approaches the theoretical limit of 4 as the data size is increased.
(TIF)

Figure S2 **Predicted top activated upstream pathway of top 250 differentially expressed genes.**
(TIF)

Figure S3 **Predicted top inhibited upstream pathway of top 250 differentially expressed genes.**
(TIF)

Table S1 **Sample list.**
(XLS)

Table S2 **Top 250 differentially expressed genes between lymphoblastoid cell line and Burkitt's lymphoma samples.**
(XLS)

Table S3 **All differentially expressed genes between lymphoblastoid cell line and Burkitt's lymphoma samples.**
(XLS)

Software S1 **Source code for RNA CoMPASS.**
(TXT)
