# Supplementary figures and images for: RNA CoMPASS: A Dual Approach for Pathogen and Host Transcriptome Analysis of RNA-Seq Datasets
Source: PLoS One. 2014 Feb 25;9(2):e89445. doi: 10.1371/journal.pone.0089445 (PMC3934900; doi:10.1371/journal.pone.0089445)

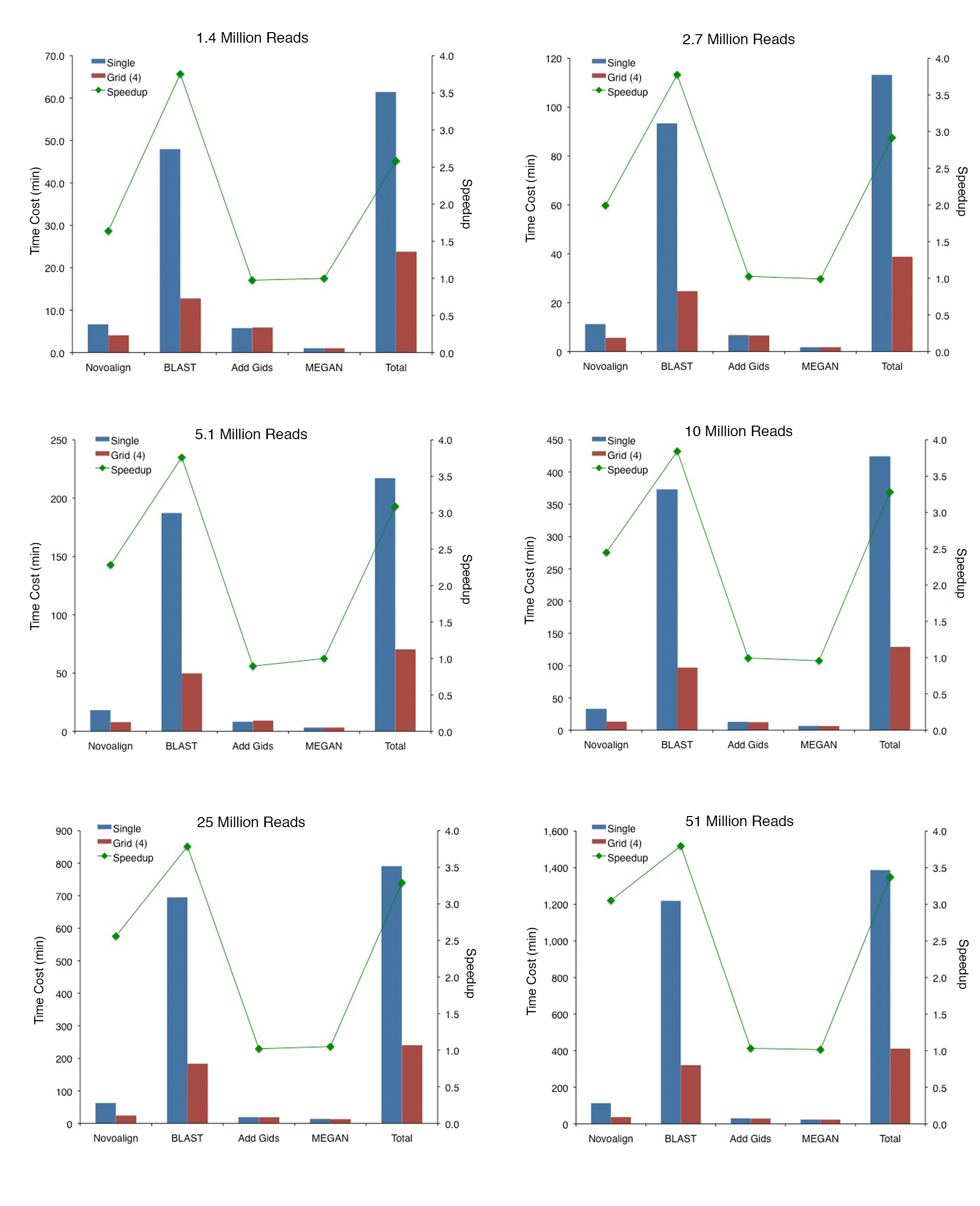

Supplement: Figure S1 — Performance of RNA CoMPASS based on individual tasks. The six Akata RNA-seq data set files used previously were benchmarked on completion of individual tasks and represented in the graphs. Runs on a single node are represented using blue columns while runs on a 4-node cluster are represented using red columns. The green line represents speedup time between the single node and 4-node environment. Note in particular that speedup of the BLAST portion of RNA CoMPASS and overall speedup approaches the theoretical limit of 4 as the data size is increased. (TIF) [file pone.0089445.s001.tif]

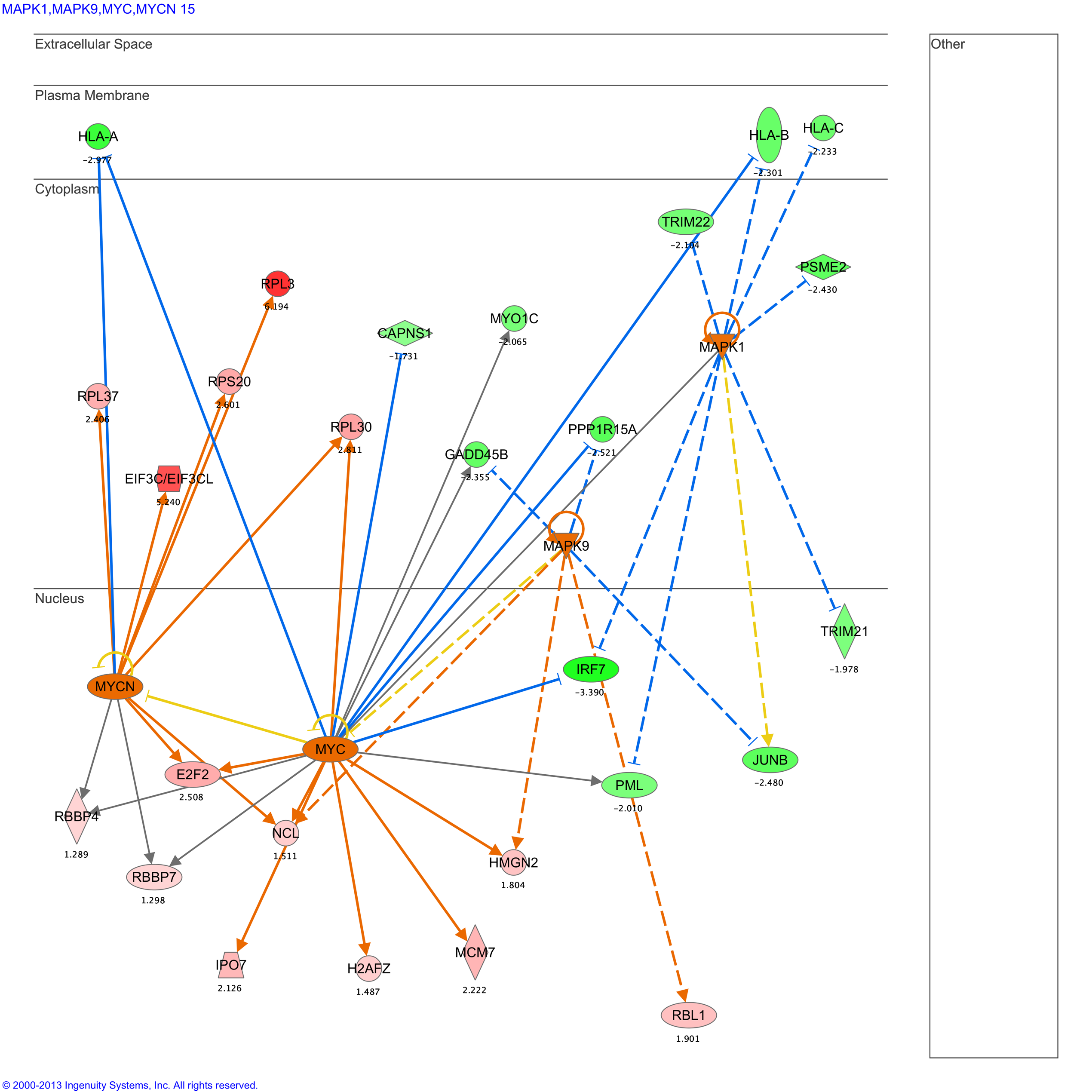

Supplement: Figure S2 — Predicted top activated upstream pathway of top 250 differentially expressed genes. (TIF) [file pone.0089445.s002.tif]

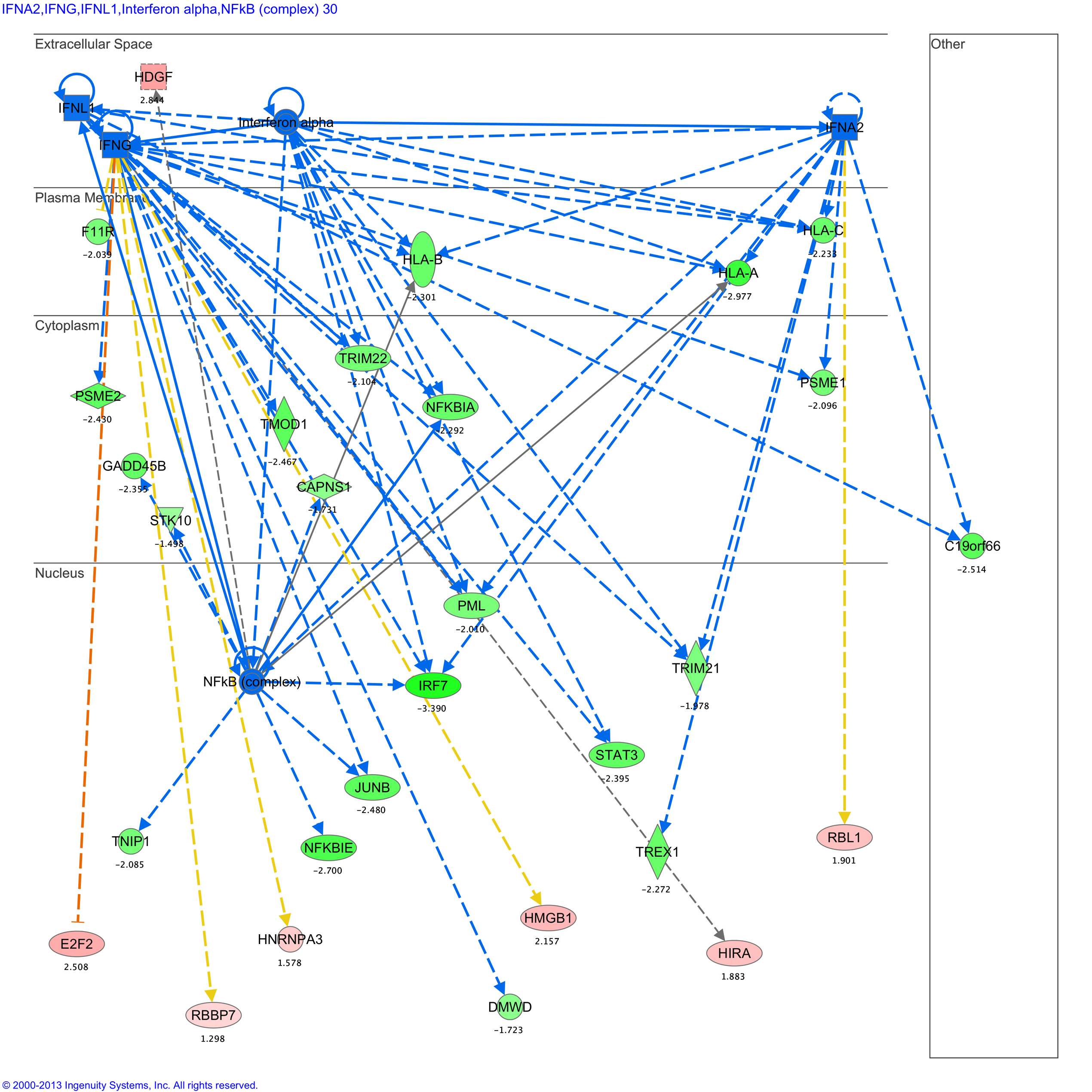

Supplement: Figure S3 — Predicted top inhibited upstream pathway of top 250 differentially expressed genes. (TIF) [file pone.0089445.s003.tif]
